# Characteristics of multicystic biliary hamartoma: A case report

**DOI:** 10.3389/fsurg.2022.1074899

**Published:** 2023-01-06

**Authors:** Jia Lian, Lixia Sun, Yankai Yang, Jun Li, Ye Zhang, Guiqiu Liu, Weijuan Hu

**Affiliations:** ^1^ Department of Gastroenterology and Hepatology, The Third Central Hospital of Tianjin, Tianjin, China; ^2^ Department of Gastroenterology and Hepatology, The Third Central Clinical College of Tianjin Medical University, Tianjin, China; ^3^ Tianjin Key Laboratory of Extracorporeal Life Support for Critical Diseases, Tianjin, China; ^4^ Artificial Cell Engineering Technology Research Center, Tianjin, China; ^5^ Tianjin Institute of Hepatobiliary Disease, Tianjin, China; ^6^ Department of Hepatobiliary Surgery, The Third Central Hospital of Tianjin, Tianjin, China; ^7^ Department of Pathology, The Third Central Hospital of Tianjin, Tianjin, China; ^8^ Department of Radiology, The Third Central Hospital of Tianjin, Tianjin, China

**Keywords:** liver, cystic lesion, hamartoma, multicystic biliary hamartoma, operation

## Abstract

**Introduction:**

Multicystic biliary hamartoma (MCBH) is a very rare hepatic benign neoplasm that manifests as a localized cystic-solid mass. Only 17 cases have been described in the literature to date. MCBH diagnosis is currently dependent on imaging and pathology following surgical resection and no precise standards are in place.

**Case Presentation:**

This case study involves a middle-aged male patient with a history of drinking but no other liver diseases. A routine ultrasound examination showed a 6.0 × 5.5 cm inhomogeneous echo mass in the right lobe of the liver. The patient experienced no discomfort or other symptoms, and blood tests were normal. Imaging revealed a localized cystic-solid neoplasm in segment 6 of the liver that did not have the features of a malignant tumor. Surgical resection was performed. Based on imaging, macroscopic examination, and histological results, a final diagnosis of MCBH was made.

**Conclusion:**

The imaging and pathological features of MCBH were summarized based on the published case reports to date. As a non-invasive examination, the imaging features will aid in the diagnosis of MCBH. Furthermore, these features, along with tumor size and patient symptoms, will facilitate clinicians in selecting surgical resection or follow-up for individual patients.

## Introduction

Patients with hepatic neoplasm are frequently encountered in our clinic. Common causes include hepatocellular carcinoma, cholangiocarcinoma, secondary malignant liver tumors, hemangioma, and abscesses. Diagnosis is dependent on whether there is chronic liver disease, a history of malignant tumors, positive tumor markers, particular imaging features, and pathological manifestations. Some rare hepatic cystic lesions, including mesenchymal hamartoma (HMH), Von Meyenburg's complex (VMC), Caroli's disease, Biliary cystadenoma, ciliated hepatic foregut cyst (CHFC), intraductal papillary neoplasm of the bile duct (IPNB), are also encountered.

Multicystic biliary hamartoma (MCBH) is a very rare hepatic localized cystic–solid neoplasm. MCBH diagnosis is still based on the imaging and pathological characteristics of surgically resected specimens due to a lack of characteristic diagnostic criteria in imaging, effective serological markers, or genetic detection. This case study in a 44-year-old male patient describes the eighteenth reported case of MCBH. Since this was considered a focal benign neoplasm, surgical resection was performed. A diagnosis of MCBH was made using a combination of imaging, macroscopic examination, and histological results.

In 2010, Ryu Y et al. (1) first described the imaging features of MCBH. In the present report, all published cases of MCBH to date were reviewed, and the imaging and histological features of MCBH were summarized. Importantly, this case report focused on imaging characteristics that would aid the diagnosis of MCBH by non-invasive examination.

## Case report

This case was a 44-year-old male patient. During his routine physical examination, an approximately 6.0 × 5.5 cm inhomogeneous echo mass was found incidentally in the right lobe of the liver by abdominal ultrasound. The patient denied any accompanying symptoms such as anorexia, abdominal distension or pain, fever, or weight loss. He had a 5-year history of hypertension and took felodipine tablets to control blood pressure. The patient had a 20-year drinking history, equivalent to about 40 g ethanol/day. He had no history of intravenous drug use, exposure to herbal medicines or health care products, or surgical and familial genetic disease. Routine blood analysis was conducted and the values for various tests—liver and kidney function, coagulation function, and tumor markers (alpha-fetoprotein, carcinoembryonic antigen, and carbohydrate antigen 19-9)—were within the normal range. Serological tests for hepatitis B and hepatitis C were negative. Autoantibodies related to autoimmune liver disease, thyroid function, and ceruloplasmin were within the normal range. Physical examination showed no positive disease indicators.

**Figure 1:**
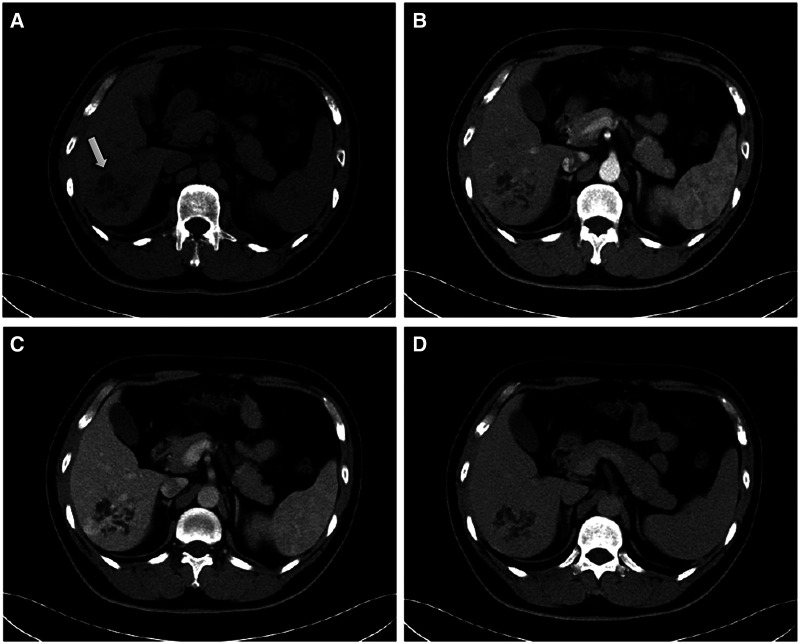
Contrast-enhanced CT findings. (**A**) plain scan, (**B**) arterial, (**C**) portal and (**D**) equilibrium phases, respectively. The CT scan shows a honeycomb-like cystic–solid lesion in segment 6 of the liver (white arrow). The cystic components show no enhancement during the arterial phase. The solid components are more enhanced compared with the normal hepatic parenchyma in the arterial and portal phases and are consistent with the normal hepatic parenchyma in the equilibrium phase.

**Figure 2:**
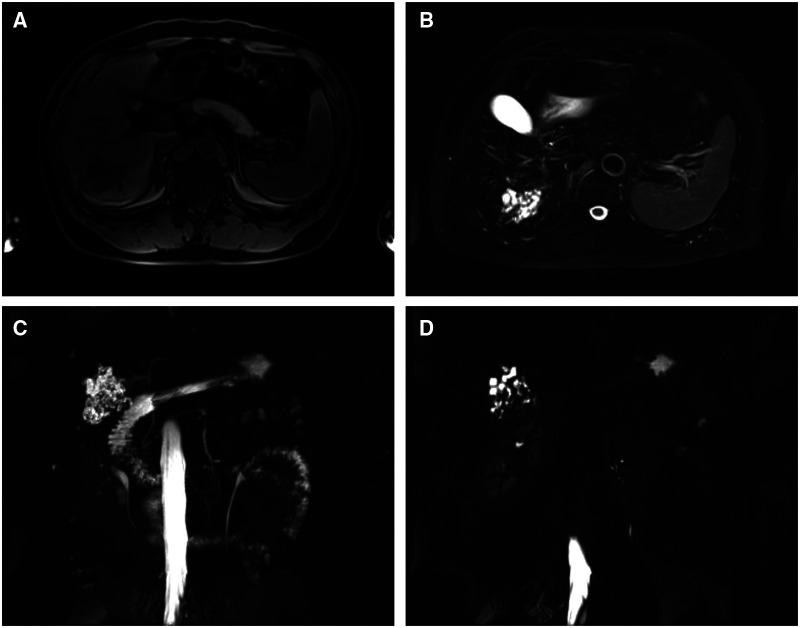
MRI findings of a lesion that is an irregular tubular (**A**) low-density area on T1 and (**B**) high-intensity area on T2. (**C**) and (**D**) MRCP findings of the mass that does not communicate with the bile duct. The intrahepatic and extrahepatic bile ducts are not dilated.

The patient then underwent an imaging examination. A second ultrasound revealed multiple small irregularly shaped hypoechoic masses with slightly hyperechoic septae in segment 6 of the liver (S6), and a total size of approximately 6.0 × 5.5 cm. Contrast-enhanced ultrasound (CEUS) showed a cystic-solid lesion with honeycomb-like enhancement in the arterial phase, in which multiple disordered unreinforced tubular columnar areas were seen. No obvious papillary structure was found. The enhanced region was slowly cleared in the portal and delayed phases. Abdominal contrast-enhanced computed tomography (CT) scans (Figure 1) showed a honeycomb-like cystic-solid lesion with a tubulocystic manifestation lacking well-defined borders in S6 and no dilation of the major intrahepatic bile duct in the background liver. The cystic components were low-density and showed no enhancement in the arterial phase. The solid components, which were septa or the cystic wall, were more enhanced than the normal hepatic parenchyma in the arterial and portal phases and were consistent with normal hepatic parenchyma in the equilibrium phase. Abdominal magnetic resonance imaging (MRI) showed an irregular-shaped multicystic mass with a mixed signal shadow in S6. The lesion was revealed as an irregular tubular low-density area on T1-weighted images and a high-intensity area on T2-weighted images, which were interspersed with strips of slightly higher signal shadows. The signal of the solid component of the intermediate inclusion was not high on diffusion-weighted imaging (DWI), while the apparent diffusion coefficient (ADC) signal was high, indicating that the dispersion was not limited. The solid components of the lesion were enhanced in the late arterial phase by injecting the contrast medium, gadoxetic acid disodium. In the hepatobiliary phase, the whole lesion was low signal. The mass had no obvious invasion into adjacent structures and was thought to be benign. Magnetic resonance cholangiopancreatography (MRCP) showed intrahepatic hybrid-density cystic-solid masses that did not communicate with the bile duct. Intrahepatic and extrahepatic bile ducts were not dilated. No definite abnormal signal shadow was found in the bile duct cavity and gallbladder. Imaging examination did not reveal any bile duct stones (Figure 2). To exclude liver metastatic carcinoma caused by gastrointestinal malignancies, gastroscopy and colonoscopy were performed and no obvious abnormalities were detected. Duodenal papilla was normal, and no colloidal mucus was present. Based on these results, the lesion was suspected to be MCBH but other diseases such as HMH, VMC, Caroli's disease, biliary cystadenoma, and CHFC could not yet be excluded.

Since MCBH is a localized cystic-solid lesion, it can be difficult to diagnose by needle biopsy due to limited sampling of the lesion and heterogeneous distribution of the tumor components. After communicating with the patient and his relatives, surgical resection was performed. This was an open operation. The lesion could not be observed in the liver surface. The intraoperative ultrasonic testing was performed and the lesion was located in the right posterior segment VI of liver. Anatomical resection of segment VI was performed and the resection margin was more than 1 cm to the lesion. No enlarged lymph nodes were found during the operation. The residual liver had no tumors and showed healthy texture by intraoperative ultrasound. The operation was successful, lasting about 2 h, and the intraoperative bleeding was 100 ml.

The surgical specimen revealed an approximately 6.0 × 5.5 cm nodular mass. A cystic-solid lesion with a honeycomb appearance and gray-white, medium texture, was seen in a section of the resected specimen. The lesion was composed of diffuse, cystically dilated ductal structures that were approximately 0.1–1.5 cm in diameter and surrounded by fibrous tissue. The lesion was filled with clear, colorless liquid surrounded by normal liver tissue.

**Figure 3:**
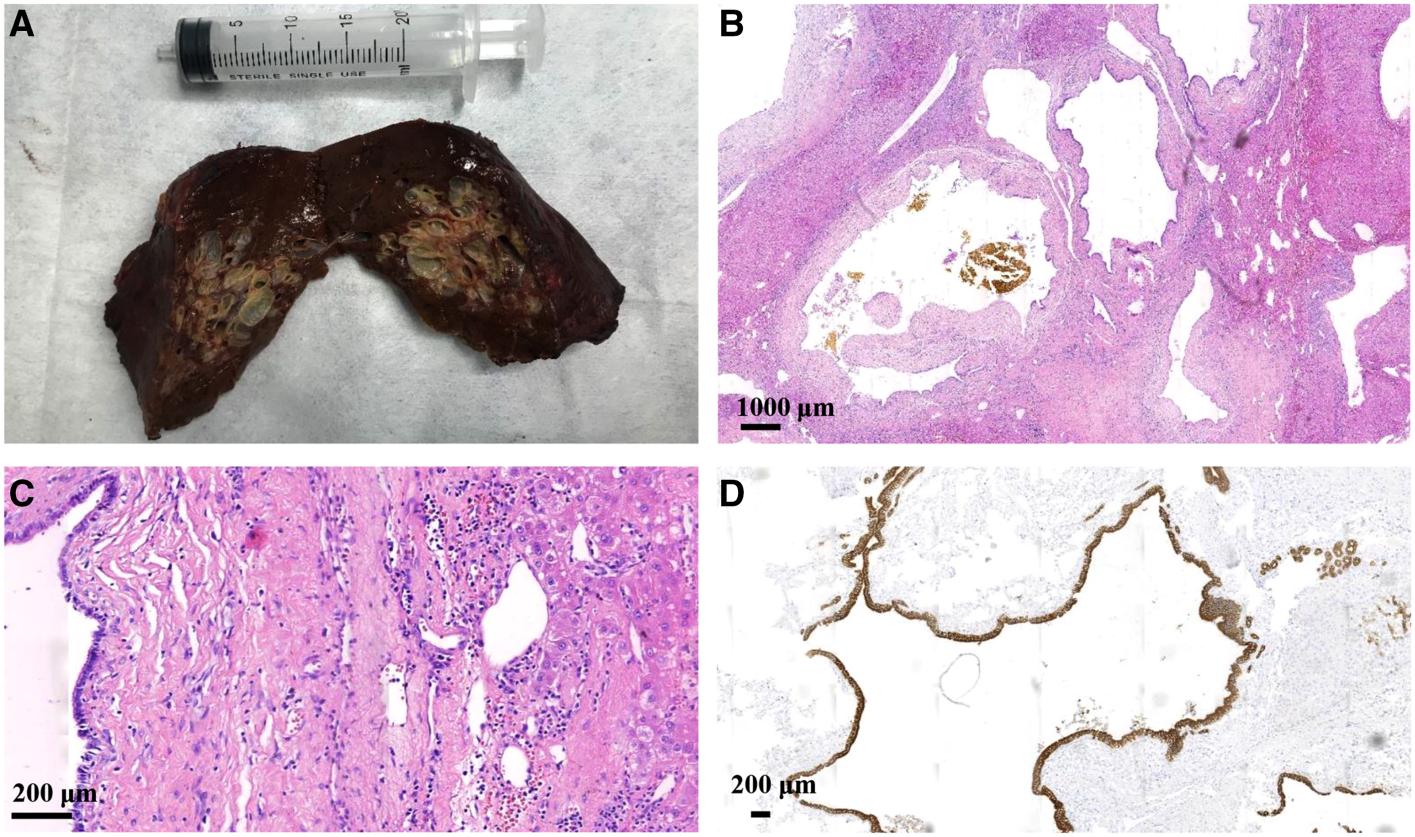
The surgical specimen (**A**) shows a nodular mass with a gray-white honeycomb-like cystic-solid lesion observed in one section. Hematoxylin and eosin staining under (**B**) low-power microscopy displaying cystically dilated ductal and irregularly angulated structures along with bile-stained materials observed in some ducts, and (**C**) high-power microscopy showing that the ductal epithelium is composed of monolayered columnar and cuboidal epithelium. Normal hepatocytes are observed between the cystic ducts. (**D**) Immunohistochemistry showing CK7 and CK19 positivity in the dilated duct epithelium.

Low-power microscopy displayed a relative clearance boundary in the lesion area that consisted of ductal structures, periductal glands, fibrous connective tissues, and blood vessels. Ductal structures were cystically dilated and irregularly angulated. Bile-stained materials were observed in some ducts and the peripheral bile ducts were not dilated. High-power microscopy showed that the ductal epithelium was composed of a monolayered columnar and cuboidal epithelium that was morphologically identical to biliary epithelium. Fibrous connective tissue around the ducts contained only mild lymphocytic infiltration. Normal hepatocytes were observed between the cystic ducts. There were no smooth muscle elements or ovarian-like stroma, and there were no atypical cells or papillary growth of the epithelial cells. Synchronous biliary hamartomas, nodules, steatosis, or significant fibrosis were not observed in the non-lesion liver tissue. Immunohistochemistry showed CK7 and CK19 positivity in the dilated duct epithelium and CD34 positivity in the vessels. Ki-67 antigen staining revealed the proliferative activity of individual cells (Figure 3).

## Final diagnosis

Based on the clinical manifestations, imaging and histological results, the final diagnosis was confirmed as MCBH. The patient recovered well after the operation and was discharged from the hospital. At 6 mo postoperatively, the patient was still alive.

## Discussion

MCBH is a very rare hepatic benign neoplasm. It was first reported in 2005 (2) and described as a solitary cystic lesion of bile duct hamartoma. Zen et al. (3) proposed the concept of MCBH and described its characteristic pathological findings. Kai K et al. (4) reported a case with an intrahepatic lesion type, and suggested that the lesion occurred not only on the liver surface but also within the hepatic parenchyma. In 2010, Ryu Y et al. (1) first summarized the imaging features of MCBH. The lesion described in the current case report was near the liver surface but did not protrude outside. Based on Zen et al.'s standard and Ryu et al.'s imaging features, this lesion was diagnosed as MCBH. However, there were still several other diseases that needed to be ruled out, including HMH, VMC, Caroli's disease, Biliary cystadenoma, CHFC, and IPNB.

HMH is a large, well-circumscribed, multiloculated cystic mass (5) that can vary in size up to >30 cm. The cystic structures contain yellowish fluid with occasional gelatinous material (6). Most (80%) HMH patients are ≤2 years of age (7). Very few adult cases are reported, and female incidence is relatively higher. The patient described in the current study was a middle-aged man. He had a cystic-solid lesion without well-defined borders and had cystically dilated ductal structures measuring 0.1–1.5 cm in diameter that were filled with clear, colorless liquid. These findings, combined with the pathology, do not support a diagnosis of HMH.

VMC is characterized by discretely distributed, well-defined, cystically dilated bile ducts. Contrary to the relatively large size of nodules in MCBH, the nodules in VMC are small (<1.5 cm), usually between 0.2 and 0.5 cm in diameter. No enhancement is seen using enhanced CT/MRI (8). The current case was a focal lesion. The cystically dilated ductal structures were 0.1–1.5 cm and most were >0.5 cm. The solid components were enhanced using contrast agents. The current case did not support a VMC diagnosis, but further verification is needed to rule out the possibility that it is a VMC variant.

The typical imaging manifestation of Caroli's disease includes enlarged intrahepatic bile ducts that communicate with the bile duct system (9), and accompanied by a “central dot sign” (10). The lesions are not enhanced after contrast injection and are often accompanied by congenital hepatic fibrosis. The current case had no “central dot sign” and the solid components were enhanced in the arterial phase. The cystic dilatation tubes were not linked to the bile duct, and there was no hepatic fibrosis. Thus, Caroli's disease can be safely excluded.

Biliary cystadenoma is rarely encountered in males. The condition manifests as multiple septa in the large cyst which are divided into multiple small cysts of different sizes, usually with “ovarian-like” stroma. Imaging of the patient in the current case study revealed multiple tubular shadows twisted into a honeycomb-like lesion with solid components in the middle. There were clear differences in the imaging manifestations of these two diseases. In addition to the lack of “ovarian-like” stroma, the current case did not support biliary cystadenoma. CHFC has a unilocular cystic appearance and the presence of a four-layered cyst wall (11). A ciliated columnar epithelium is essential for the diagnosis of CHFC. Thus, this disease can be excluded.

IPNB is characterized by marked dilation or cystic lesions of the bile ducts with papillary structures (12) which are connected to the main hepatic duct, and the duodenal papilla is usually accompanied by colloidal mucus outflow. The current patient's liver function and tumor markers were normal, imaging did not reveal any papillary structures, and the lesions did not communicate with the biliary system. Gastroscopy revealed no colloidal mucus overflow from the duodenal papilla. Thus, this disease can also be excluded.

All published literature on MCBH were collected. Only 17 cases have been recorded in the literature to date, with the one described here being the eighteenth case (Table 1). MRI of the current case revealed that the DWI signal was not high while the ADC signal was high. There was no obvious invasion of adjacent structures, so the possibility of a malignant lesion was essentially ruled out. After reviewing all 18 case reports, the characteristics of MCBH are summarized as follows: (1) A neoplasm generally located near the liver surface and/or protruding from the liver; (2) A localized cystic–solid neoplasm with a honeycomb-like appearance without well-defined borders. The cystic components show no enhancement, while the solid components are enhanced in the arterial phase; (3) Intrahepatic and extrahepatic bile ducts are not dilated. The cystic dilatation tubes are not connected to the biliary system; (4) The neoplasm is composed of ductal structures, periductal glands, and fibrous connective tissues, and normal liver parenchyma intermingles within the nodular lesion; (5) The neoplasm contains bile-like materials within ducts; (6) Biliary-type CKs are positive on immunostaining. Given the patient's age, sex, previous disease history, blood test results, and 1–3 imaging characteristics, many diseases, including HMH, VMC, Caroli's disease, Biliary cystadenoma, and IPNB, could be excluded. However, a small number of diseases require diagnosis through pathology. The imaging features described here should help to narrow the scope of differential diagnosis and aid early identification and diagnosis of MCBH.

**Table 1:** Summary of cases with multicystic biliary hamartoma (MCBH).

Case No.	Ref.	Age (years)/Sex	Size (cm)	Location	protruding from the liver	Imaging or histological features
1	Kobayashi et al, 2005 (2)	30/M	3.6	Seg VI	No	Small cysts (<0.5 cm) and larger cysts (0.5–1.2 cm), lined by low columnar or cuboidal epithelium, contained bile-stained material, were embedded in a fibrous stroma
2	Zen et al, 2006 (3)	59/M	4.2	Seg VI	Yes	A relatively well-circumscribed nodule was enhanced on CT by contrast medium and sustained until the delayed phase. Histologic features included xanthogranulomatous inflammations, ductal structures, periductal glands, and fibrous connective tissues containing blood vessels. Some ducts contained bile-like brown materials with focal calcifications. Ductal epithelium diffusely expressed biliary-type CKs such as CK7 and CK19
3		70/F	1.8	Seg III	Yes	CT (arterial phase) displays multiple tiny cysts with thin walls and septae. Walls and septae are enhanced slightly. CT (equivalent phase) cyst walls and septae are more markedly enhanced than in arterial phase. CT during hepatic arteriography (CTHA) clearly shows enhanced area of the cysts wall and septae. Cut surface demonstrates honeycomb-like appearance
4		69/F	2.8	Seg III	Yes	A multilocular cystic lesion containing many small cystic spaces
5	Kai et al, 2008 (4)	55/M	5.0	Seg VI	No	CT imaging during arterial portography (CTAP) showed an intrahepatic, multicystic, honeycomb-like lesion with contrast enhancement in a part of the septum. A low density area on T1-weighted images and a multiple bulboid high intensity area on T2-weighted images. The bile ducts were not dilated in the background liver
6	Ryu et al, 2010 (1)	45/M	2.0–3.5 (cases no. 6-8)	Seg VII	No	MRI and CT scan showed normal liver parenchyma was intermingled around the lesion. Pathological findings demonstrated hepatic parenchyma was observed among ductal structures
7		58/M		Seg III	No	CT (immediately after endoscopic retrograde cholangiography) showed that contrast medium did not enter the lesion
8		55/F		Seg VI/VII	No	
9	Song et al, 2013 (13)	52/M	2.7	Seg III	No	On MRI, MCBHs are hypointense on T1-weighted imaging. T2-weighted imaging reveals a multicystic, honeycomb-like lesion with bright, high signal intensity. Histologically, the lesion consisted of multiple dilated cystic ducts lined by biliary type epithelial cells, periductal glands and connective tissue, which included small amounts of hepatic parenchyma and blood vessels
10	Beard et al, 2014 (14)	48/F	4.7	Seg VIII	No	The radiographic appearance is that of a peripherally located, tubulocystic, honeycomb-like mass, consistent with an aggregate of dilated biliary ducts and intermingled normal hepatic parenchyma. The more common occurrence at the periphery rather than centrally. Pathological findings included microscopic islands of hepatic parenchyma within fibrous tissue that were not just at the periphery, but also in the center of the lesion
11	Yoh et al, 2014 (15)	69/M	3.0	Seg III	No	The peripheral site of this lesion is slightly enhanced on the arterial phase. On the portal phase, the ring-enhancement of the lesion is clearer and shows honeycomb-like dilated bile duct
12	Fernández-Carrión et al, 2015 (16)	60/F	5.0	Seg VI	No	
13	Tominaga et al, 2015 (17)	26/M	10.0	Seg V/VI	Yes	MRCP revealed an intrahepatic cystic tumor which did not communicate with the main hepatic duct in the hilum. The left bile duct and the common hepatic duct were not dilated, and there was no communication with the main duct
14	Morinaga et al, 2017 (18)	53/M	12.0	left lobe of the liver	Yes	Positron-emission tomography (PET)-CT revealed no uptake of fluorodeoxyglucose in the lesion. On endoscopic retrograde cholangiopancreatography (ERCP), the multicystic lesion did not communicate with the main hepatic bile duct
15	Ogura et al, 2018 (19)	77/F	12.0	Seg III	Yes	No communication between the biliary tract and the lesion is evident under ERCP
16	Mu et al, 2021 (20)	37/M	7.7	Seg VI	No	
17	Wang et al, 2022 (21)	14/M	17.0	Seg III	No	
Present case		44/M	6.0	Seg VI	No	

## Conclusions

MCBH is a very rare hepatic benign neoplasm that is associated with a localized cystic-solid mass. The incidence and natural history of this disease remain unknown. In the absence of characteristic diagnostic imaging criteria, effective serological markers, or genetic detection, diagnosis is dependent on imaging combined with histology after surgical resection. In this case study, we summarize the imaging and histological features of this disease. Importantly, we focus on those imaging characteristics that aid the diagnosis of MCBH using non-invasive methods. Imaging results combined with neoplasm size and patient symptoms will facilitate clinicians in selecting surgery or follow-up for individual patients, thereby preventing the need to rely on simple surgical resection and consequently reducing pain and economic burden for patients.

## Data Availability

The raw data supporting the conclusions of this article will be made available by the authors, without undue reservation.
